# Analysis of Digital Ischemic Necrosis from Constrictive Finger Dressings: A Case Series

**DOI:** 10.1055/a-2749-5565

**Published:** 2026-01-30

**Authors:** Dong Chul Lee, Sung Hyun Hwang, Kyung Jin Lee, Sung Hoon Koh, Si Young Roh, Jin Soo Kim

**Affiliations:** 1 Department of Plastic and Reconstructive Surgery, Gwangmyeong Sungae General Hospital, Gwangmyeong, Korea

**Keywords:** digital ischemia, necrosis, iatrogenic injury, free tissue flaps

## Abstract

Digital ischemic necrosis can result from circumferential compression by medical dressings or domestic materials, particularly in pediatric patients. We present nine cases of ischemic necrosis caused by constrictive finger dressings between 2014 and 2024. Seven cases were iatrogenic, related to medical dressings, and two were caregiver-induced. Caregiver-induced cases tended to show more severe injuries, often necessitating reconstructive procedures such as free flaps, and these patients generally presented later than those with iatrogenic injuries. The contrast in timing and severity between the two groups suggests that delayed recognition may contribute to less favorable outcomes. These cases highlight the preventable nature of this condition and emphasize the importance of early recognition, proper wound monitoring, and caregiver education to minimize tissue loss and reduce the need for complex reconstructive surgery.

## Introduction

Digital ischemic necrosis results from prolonged vascular compromise that leads to irreversible tissue damage. Circumferential compression of the finger is a particularly important cause, as its cylindrical morphology makes it vulnerable to impaired circulation. 1 2 3 Both medical dressings, such as Coban™ (3M Nexcare; MN) or tubular gauze, and everyday objects like rubber bands or band-aids have been implicated in such injuries. 4 5 Although these devices normally exert uniform pressure on the affected area, the rolling process may inadvertently lead to excessive focal pressure, potentially compromising local circulation. 6 Iatrogenic cases often occur after improper application of dressings, whereas caregiver-induced injuries may result from unawareness of circulatory risks. 4 Delayed recognition can worsen tissue loss and necessitate reconstructive surgery. 7 8 Although individual cases have been reported, series describing multiple patients are rare. Here, we describe nine patients with digital ischemic necrosis from constrictive finger dressings, emphasizing their clinical features, management, and preventive implications. Institutional Review Board (IRB) approval of our institution was obtained (IRB number 2025-N-001), and all patient data were anonymized. All participants provided written informed consent for publication, including clinical images.

## Case

Between August 2014 and August 2024, nine patients were treated at our institution for digital ischemic necrosis caused by circumferential compression. Seven cases were iatrogenic following the application of medical dressings, most commonly Coban™, and two cases were caregiver-induced, involving a rubber band and a hair tie. Pediatric patients were predominantly affected, accounting for seven of the nine cases. The depth of necrosis varied from superficial dermal involvement to full-thickness loss with exposure of the distal phalanx. The extent of injury varied from fingertip or nailfold-level lesions to involvement reaching the distal interphalangeal joint (DIPJ) or even the proximal interphalangeal joint.

In the iatrogenic group, compression was typically applied for hemostasis or splinting. Patients presented after an average delay of 5.5 days. Management ranged from conservative observation to full-thickness skin grafts and, in one case, free flap reconstruction. In the caregiver-induced group, patients presented later, with a mean delay of 7.3 days, and exhibited more extensive necrosis. Both required free flap coverage. In cases with osseous involvement and flap reconstruction, residual sequelae such as digital length shortening and sensory dullness were observed during follow-up. Tables 1 and 2 summarize all nine patients, including age, cause, treatment, and outcomes.

**Table 1:** Patient demographics

Case	Age	Sex	Injury type	Compression material	Time to presentation
1	58 years	F	Skin defect	Coban	2 days
2	13 months	M	Laceration	Coban	3 days
3	15 months	M	Closed fracture	Coban	7 days
4	35 years	F	Laceration	Unspecified	10 days
5	43 years	F	Laceration	Coban	6 days
6	3 years	F	Open fracture	Coban	3 days
7	13 months	M	Laceration	Coban	3 days
8	6 years	F	Puncture wound	Rubber band	4 days
9	6 years	F	No trauma	Hair tie	8 days

**Table 2:** Summary of injury severity, reconstructive approaches, and prognosis

Case	Depth	Range	Treatment option	Clinical outcome
1	Dermis	DIPJ level	FTSG	Scar
2	Dermis	Proximal phalanx shaft	Observation	Scar
3	Distal phalanx bone	Ulnar half Middle phalanx shaft	Hypothenar free flap	Digital length shortening
4	Dermis	PIPJ level	Observation	Scar
5	Dermis	Nailfold level	FTSG	Scar
6	Dermis	Fingertip	FTSG	Scar
7	Dermis	Radial half Fingertip to nail fold	FTSG	Scar
8	Distal phalanx bone	Nailfold level	Second TPFF	Digital length shortening Dullness
9	Distal phalanx bone	Nailfold level	Second TPFF	Digital length shortening Dullness

Abbreviations: DIPJ, distal interphalangeal joint; FTSG, full-thickness skin graft; PIPJ, proximal interphalangeal joint; second TPFF, second toe pulp free flap.

### Case 1

**Fig. 1:**
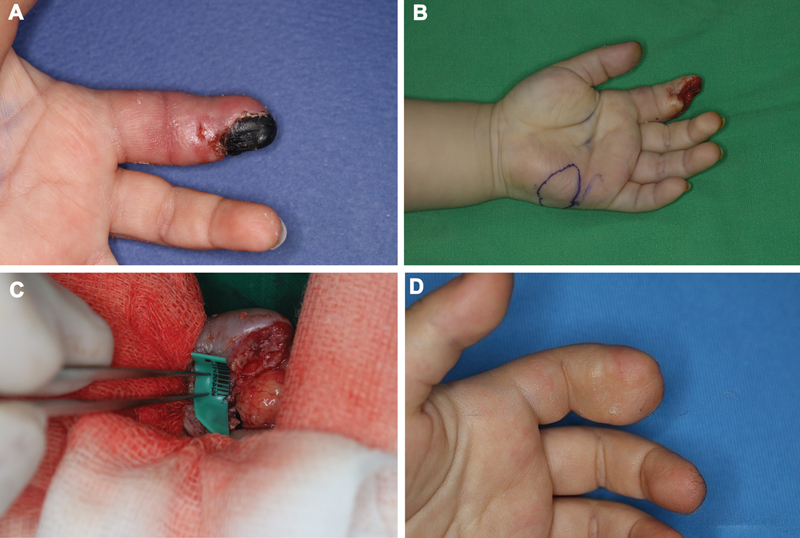
( **A** ) A 15-month-old male sustained an injury to his index finger. ( **B, C** ) Soft tissue coverage by hypothenar muscle perforator free flap. ( **D** ) The 24-month postoperative follow-up appointment.

A 15-month-old boy presented with necrosis of the left index finger after a Coban™ was applied as a splint for a tuft fracture. The dressing was left in place for 7 days before necrosis became evident. Physical examination showed color changes and complete sensory loss distal to the middle phalanx ( Fig. 1A ). Doppler ultrasonography revealed no flow signals in the demarcated zone. The necrotic portion was amputated, and a hypothenar muscle perforator free flap was performed ( Fig. 1B, C ). The flap survived, although minor wound dehiscence required secondary closure. At 2-year follow-up, the patient demonstrated sequelae, including a limited range of motion and discomfort associated with the reconstructed digit ( Fig. 1D ).

### Case 2

**Fig. 2:**
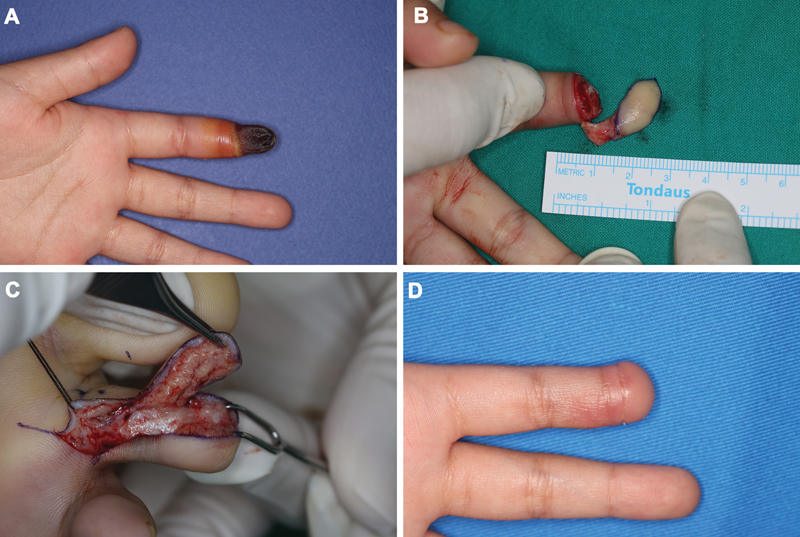
( **A** ) A 6-year-old female with a necrotic change on her index finger. ( **B, C** ) Soft tissue coverage by a second toe pulp free flap. ( **D** ) Four-week postoperative follow-up photograph.

A 6-year-old girl had a rubber band around her finger by her mother for 4 days to control bleeding from a minor wound. She presented with necrosis extending to the distal interphalangeal joint. Initial conservative management with topical antibiotics and observation failed to halt progression, and amputation was performed at the DIPJ ( Fig. 2A ). Reconstruction with a second toe pulp free flap was carried out ( Fig. 2B, C ). The flap healed uneventfully, but functional limitations related to joint loss were anticipated at follow-up ( Fig. 2D ).

## Discussion

Digital ischemic necrosis can arise from both medical dressings and domestic objects, as demonstrated in our series. Iatrogenic cases were typically caused by Coban™ or other self-adherent bandages applied for hemostasis or immobilization. In contrast, caregiver-induced injuries from objects such as rubber bands tended to present later and with more extensive necrosis.

### Pediatric Vulnerability

In our series, digital necrosis was observed predominantly in pediatric patients, suggesting increased vulnerability in this population. Children may not verbalize discomfort, leading to delayed recognition. Moreover, given the smaller size of pediatric digits, even the same compression technique may result in disproportionately higher pressure, potentially increasing the risk of ischemic injury and necrosis. Therefore, proper monitoring for symptoms such as pain or sensory loss and signs of impaired circulation is crucial. 9 10 Moreover, when applying compression dressings in pediatric patients, it is essential to provide thorough education to both the patients and their caregivers.

### Material Impact

Compression dressing materials, such as Coban™, are commonly used to achieve hemostasis and stabilization. 6 However, it has been suggested that pressures of 200 to 300 mm Hg applied on the upper arm or forearm for 2 hours are sufficient to cause neurovascular and muscular damage, while pressures below 100 mm Hg can still induce ischemic changes if maintained for extended durations. 11 In digits, smaller vasculature and limited collateral circulation render them even more vulnerable to ischemic injury under similar conditions because of disproportionately higher pressures to their reduced radius.

For example, Coban™ has been documented to generate pressures exceeding 60 mm Hg, sufficient to induce ischemia over time by strangulation and eventual necrosis, especially in small digits. 12 This was especially evident when circumferential tension was unevenly distributed, causing pressure levels to vary significantly along the surface of the finger. 4 Similarly, in domestic caregiver-induced cases, everyday objects such as Band-Aids (Johnson & Johnson, New Brunswick, NJ), hair ties, and rubber bands have been previously reported to cause digital ischemic injury. 13 At our institution, domestic injuries caused by hair ties or rubber bands were observed, resulting from prolonged, unnoticed compression.

### Clinical Implication

Prolonged ischemia exacerbates tissue damage, making early detection critical for a favorable prognosis. Circulation can be assessed through capillary refill tests, while the formation of bullae and changes in digit coloration can indicate potential injury. Doppler ultrasound can further assist in the evaluation. Early identification of ischemia may allow for non-surgical interventions, such as medical leech, dressings with heparin, or topical or intravenous vasodilators such as enoxaparin, heparin, and aspirin. 14 Additionally, techniques to enhance local circulation, such as the application of warming blankets or heat lamps, have been suggested to improve outcomes. However, late presentations often require more invasive treatments, including amputation and reconstruction.

### Preventive Strategies

For patients with extensive injuries that require digital amputations, discomfort and functional impairment due to shortened length is anticipated. Moreover, reconstruction surgery typically requires extensive hospitalization, leading to substantial medical expenses. To prevent such severe outcomes, it is essential to implement appropriate preventive strategies targeting both health care institutions and caregivers.

Medical professionals must ensure that the dressing application allows circulation monitoring. First, patients receiving compression dressings should be advised to visit the hospital daily or every 2 days, allowing for close monitoring of any vascular compromise. Moreover, fingertips need to be exposed to facilitate circulation monitors. When applying bandages, care should be taken to distribute pressure evenly from the fingertip to the wrist, avoiding excessive compression in any specific area. 4 15 Regulatory guidelines should address the risks associated with self-adherent bandages in home use.

As domestic cases in our series often involved more extensive injuries requiring free flap procedures, it appears crucial to educate caregivers. We suggest that this is due to caregivers' lack of awareness regarding digital ischemia, leading to delayed hospital presentations. Therefore, both patients and caregivers must be educated about diagnostic procedures such as the capillary refill test and how to identify warning signs of ischemia, such as paresthesia, severe pain, and color changes. They should be instructed to promptly remove the dressing and seek immediate medical attention if these symptoms occur.

### Conclusion

Digital ischemic necrosis from constrictive dressings is a serious but potentially avoidable condition. This study highlights the importance of appropriate dressing techniques and timely recognition of ischemia. Increased awareness among health care providers and caregivers may help reduce the incidence of such injuries.
